# Dataset on the interaction effects of tillage, crop rotations and residue management on overall soil quality

**DOI:** 10.1016/j.dib.2022.108456

**Published:** 2022-07-08

**Authors:** I. Gura, P.N.S. Mnkeni, C.C. Du Preez, J.H. Barnard

**Affiliations:** a Department of Agronomy, University of Fort Hare, Alice 5700, South Africa; b Department of Soil, Crop and Climate Sciences, University of the Free State, Bloemfontein 9301, South Africa

**Keywords:** Overall soil quality, Soil management assessment framework (SMAF), Soil quality index (SQI), Conservation agriculture (CA) strategies

## Abstract

The data reported in this article is related to the research article entitled “Short-term effects of conservation agriculture strategies on the soil quality of a *Haplic Plinthosol* in Eastern Cape, South Africa” [1]. Standard soil extraction procedures and experiments were used to generate the raw soil indicator data [2] and the data was interpreted using the Soil Management Assessment Framework (SMAF) algorithms to evaluate the impacts of tillage practices, crop rotation sequences and residue management systems on overall soil quality. The SMAF-soil quality index (SMAF-SQI) was used as an indicator of overall soil quality. The soil indicator scores were processed and analyzed using the JMP statistical package [3]. The SMAF-scored data is made accessible for supplementary use and for advancing the understanding of the main findings of the related research.

## Specifications Table

**Table:** 

Subject	Soil Science
Specific subject area	Conservation agriculture
Type of data	Table
How the data were acquired	Data scores were generated by processing soil data acquired by using soil standard methods in the field and in the laboratory. Standard machines such as Inductively Coupled Plasma Emission Spectrograph (ICP–OES) (Varian 710-ES), LECO (Truspec – CNS analyzer) and UV spectrophotometer were used to analyze soil extractions. Soil data was processed using Soil Management Assessment Framework (SMAF) algorithms. The SMAF-SQI was used as an indicator of overall soil quality. The data scores were analyzed statistically using JMP statistical package
Data format	Processed
Description of data collection	Soils subjected to CA strategies were sampled and analyzed in the field and in the laboratory for nine soil indicators *viz* ρb, AGS, pH, EC, P, K, SOC, MBC and BG activity. The soil indicator data was scored using SMAF interpretation algorithms into unit less scores ranging between 0 and 1. The scores for nine indicators were used to compute an overall SQI.
Data source location	Phandulwazi, Eastern Cape, South Africa (32° 39′S and 26° 55′E)
Data accessibility	Raw data is available at Mendeley Data Repository name: Mendeley Data Data identification number (DOI): 10.17632/j29vpkhwb9.1 Direct URL to data: https://data.mendeley.com/datasets/j29vpkhwb9/1
Related research article	I. Gura, P.N.S. Mnkeni, C.C. Du Preez, J.H. Barnard, 2022. Short-term effects of conservation agriculture strategies on the soil quality of a *Haplic Plinthosol* in Eastern Cape, South Africa. Soil Tillage Res 220, 105,378. https://doi.org/10.1016/j.Sc.2010.00372[1]

## Value of the Data

• The data provides the importance of interpreting the impacts of conservation agriculture (CA) strategies on overall soil quality using a SMAF-soil quality index (SMAF-SQI).
• The data set is valuable in improving the understanding of the impact of the interactions of all the three conservation agriculture (CA) strategies on overall soil quality in the short-term.
• The SMAF algorithms that were used to process data at field scale can be of use for further research by others who have interest in understanding overall soil quality at regional, national and continental scale.

## 1 Data Description

The data article provides the impacts of CA strategies *viz* tillage practices, crop rotation sequences, residue management systems and their interactions on soil quality indicator scores and overall soil quality. Data scores reported in this article were generated by processing raw soil indicator data using SMAF algorithms and the raw data can be accessed at Mendeley data repository [2]. The raw soil indicator data was generated from the analysis of the soil samples that were collected from the conservation agriculture (CA) field trial in Phandulwazi, Eastern Cape, South Africa. A split-split plot arrangement in a randomized complete block design with three replicates was used for the application of the treatments. The applied CA treatments were tillage practices [conventional tillage (CT) and no-till (NT)], crop rotation sequences [maize-fallow-maize (MFM), maize-fallow-soybean (MFS), maize-wheat-maize (MWM) and maize-wheat-soybean (MWS)] and residue management systems [crop residue retention (R_+_) and crop residue removal (R_-_)]. The tillage treatments were applied as the main plots, crop rotations as the sub plots and residue management as the sub-sub plots. Standard soil extraction and analytical methods were used to generate the raw soil indicator data. Table 1 shows the ANOVA results for the impacts of CA strategies and their interactions on soil quality indicator scores and soil quality index (SQI) of the *Haplic Plinthosol* in Phandulwazi, Eastern Cape, South Africa. Table 2 shows the effect of crop rotation sequences on SMAF scores and overall soil quality (SQI) scores of the *Haplic Plinthosol.* Table 3 shows the impact of residue management systems on SMAF scores and overall soil quality (SQI) scores of the *Haplic Plinthosol.* Table 4, Table 5, Table 6, Table 7 shows the impacts of the interactions of all the three CA strategies on the soil biological, nutritional, chemical and physical indicator scores and the overall soil quality index (SQI) of the *Haplic Plinthosol,* respectively.

**Table 1:** ANOVA data for soil quality assessment using the Soil Management Assessment Framework (SMAF): effects of two tillage practices, four crop rotation sequences, two residue management systems and their interactions on the soil quality indicators’ scores and the overall soil quality index (SQI) of the *Haplic Plinthosol* in Phandulwazi, Eastern Cape, South Africa.

	SMAF-scored Soil Quality Indicators									
Treatment	SOC	MBC	BG activity	K	P	pH	EC	AGS	ρb	SQI
0–5 cm depth										
T	*	*	ns	*	*	ns	ns	***	***	***
C	ns	*	ns	ns	ns	*	ns	ns	ns	ns
R	ns	**	ns	***	ns	ns	ns	ns	ns	ns
T x C	ns	**	ns	ns	ns	ns	ns	ns	ns	ns
T x R	ns	ns	ns	ns	ns	ns	ns	ns	ns	ns
C x R	ns	*	ns	ns	ns	ns	ns	ns	ns	ns
T x C x R	ns	*	ns	ns	ns	ns	ns	ns	ns	ns

5–10 cm depth										
T	ns	***	ns	ns	ns	ns	ns	ns	***	**
C	ns	ns	ns	ns	ns	ns	ns	**	ns	ns
R	*	ns	ns	ns	ns	*	ns	ns	ns	ns
T x C	ns	**	ns	ns	ns	ns	ns	ns	ns	ns
T x R	ns	*	ns	ns	ns	**	ns	ns	ns	ns
C x R	*	**	ns	ns	ns	ns	ns	ns	ns	ns
T x C x R	ns	ns	ns	ns	ns	ns	ns	ns	ns	ns

ns - treatment not significant at *P* = 0.05 probability level; *, **, *** - treatment significant at *P* = 0.05, 0.01 and 0.001 probability level, respectively. T – tillage; C – crop rotations; R – residue management; T x C x R – the interactions of tillage, crop rotations and residue management.

**Table 2:** Data on the impact of crop rotation sequences on the SMAF scores and overall soil quality index (SQI) scores of the Haplic Plinthosol at 0–5 cm and 5–10 cm depths.

	SMAF-scored Soil Quality Indicators									
	Biological attributes			Chemical attributes				Physical attributes		
Treatment	SOC	MBC	BG activity	P	K	pH	EC	AGS	ρb	SQI
0–5 cm depth										
MFM	0.73	0.99a	1.00	0.97	0.90	0.93ab	1.00	0.67	0.61	0.87
MFS	0.73	0.95b	1.00	0.98	0.91	0.95a	1.00	0.69	0.62	0.87
MWM	0.64	0.99a	0.96	0.99	0.90	0.89b	1.00	0.69	0.60	0.85
MWS	0.65	0.99a	1.00	0.96	0.90	0.95a	1.00	0.70	0.65	0.87
*P*-value	ns	0.01*	ns	ns	ns	0.01*	ns	ns	ns	ns
CV (%)	45.35	6.36	14.00	18.48	4.61	10.43	–	8.06	99.45	12.01

5–10 cm depth										
MFM	0.45	0.85	1.00	0.98	0.87	0.92	1.00	0.43ab	0.61	0.79
MFS	0.46	0.93	1.00	0.99	0.85	0.92	1.00	0.46a	0.62	0.80
MWM	0.40	0.85	1.00	0.98	0.85	0.88	1.00	0.33b	0.60	0.77
MWS	0.43	0.88	1.00	0.94	0.87	0.88	1.00	0.37ab	0.65	0.78
*P*-value	ns	ns	ns	ns	ns	ns	ns	0.002⁎⁎	ns	ns
CV (%)	39.82	22.11	0.69	12.79	6.44	13.86	–	54.03	99.45	8.81

Means in a column with different letters are significantly different; ns – not significant.

⁎ – significant at *P* = 0.05.

⁎⁎ – significant at *P* = 0.01; MFM – maize-fallow-maize; MFS – maize-fallow-soybean; MWM – maize-wheat-soybean; MWS – maize-wheat-soybean.

**Table 3:** Data on the impact of residue management systems on SMAF scores and overall soil quality index (SQI) scores of the *Haplic Plinthosol* at 0–5 cm and 5–10 cm depths.

	SMAF-scored Soil Quality Indicators									
	Biological attributes			Chemical attributes				Physical attributes		
Treatment	SOC	MBC	BG activity	P	K	pH	EC	AGS	ρb	SQI
0–5 cm depth										
R_+_	0.68	1.00a	0.98	0.96	0.92a	0.92	1.00	0.68	0.59	0.86
R_-_	0.69	0.97b	1.00	0.98	0.88b	0.93	1.00	0.70	0.65	0.87
*P*-value	ns	0.005⁎⁎	ns	ns	<0.001⁎⁎⁎	ns	ns	ns	ns	ns
CV (%)	32.37	4.92	9.80	13.57	3.08	7.49	–	23.09	70.40	8.01

5–10 cm depth										
*R*_+_	0.47a	0.88	1.00	0.95	0.86	0.88b	1.00	0.39	0.59	0.78
R_-_	0.41b	0.88	1.00	0.99	0.86	0.92a	1.00	0.40	0.65	0.79
*P*-value	0.021*	ns	ns	ns	ns	0.036*	ns	ns	ns	ns
CV (%)	28.34	15.75	–	9.29	4.83	10.01	–	38.59	70.40	7.06

Means in a column with different letters are significantly different; ns – not significant.

⁎ – significant at *P* = 0.05.

⁎⁎ – significant at *P* = 0.01.

⁎⁎⁎ - significant at *P* = 0.001; R_+_ – residue retention; R_-_ – residue removal.

**Table 4:** Data on the interactive effects of tillage practices, crop rotation sequences and residue management systems on the soil biological indicators’ scores of the *Haplic Plinthosol* at 0–5 and 5–10 cm depth.

	SOC				MBC				BG activity			
	CT		NT		CT		NT		CT		NT	
Treatments	R_+_	R_-_	R_+_	R_-_	R_+_	R_-_	R_+_	R_-_	R_+_	R_-_	R_+_	R_-_
0–5 cm depth												
MFM	0.61	0.73	0.81	0.77	1.00a	0.99a	1.00a	0.98a	1.00	1.00	1.00	1.00
MFS	0.75	0.68	0.78	0.71	1.00a	0.84b	1.00a	0.99a	1.00	1.00	1.00	1.00
MWM	0.44	0.57	0.75	0.80	1.00a	0.98a	1.00a	0.99a	1.00	1.00	0.84	1.00
MWS	0.58	0.66	0.77	0.60	1.00a	0.99a	1.00a	0.98a	1.00	1.00	1.00	1.00

5–10 cm depth												
MFM	0.55	0.46	0.51	0.28	0.55	0.87	0.98	0.99	1.00	1.00	1.00	1.00
MFS	0.42	0.48	0.49	0.48	0.99	0.95	0.94	0.86	1.00	1.00	0.99	1.00
MWM	0.39	0.35	0.41	0.47	0.72	0.84	0.97	0.88	1.00	1.00	1.00	1.00
MWS	0.54	0.37	0.44	0.38	0.89	0.79	0.99	0.86	1.00	1.00	1.00	1.00

CT – conventional tillage; NT – no-till; MFM – maize-fallow-maize; MFS – maize-fallow-soybean; MWM – maize-wheat-soybean; MWS – maize-wheat-soybean; R_+_ – residue retention; R_-_ – residue removal.

**Table 5:** Data on the interactive effects of tillage practices, crop rotation sequences and residue management systems on the soil nutritional indicators scores of the *Haplic Plinthosol* at 0–5 and 5–10 cm depth.

	P				K			
	CT		NT		CT		NT	
Treatments	R_+_	R_-_	R_+_	R_-_	R_+_	R_-_	R_+_	R_-_
0–5 cm dep								
MFM	1.00	1.00	0.99	0.89	0.90	0.88	0.94	0.89
MFS	1.00	1.00	0.90	1.00	0.91	0.88	0.94	0.89
MWM	0.97	1.00	0.98	1.00	0.91	0.85	0.94	0.88
MWS	0.97	1.00	0.89	0.96	0.91	0.86	0.94	0.90
5–10 cm								
MFM	1.00	1.00	0.92	0.99	0.87	0.87	0.86	0.86
MFS	1.00	0.98	0.97	1.00	0.87	0.87	0.84	0.84
MWM	1.00	1.00	0.94	1.00	0.86	0.85	0.84	0.86
MWS	0.89	1.00	0.92	0.96	0.87	0.88	0.83	0.89

CT – conventional tillage; NT – no-till; MFM – maize-fallow-maize; MFS – maize-fallow-soybean; MWM – maize-wheat-soybean; MWS – maize-wheat-soybean; R_+_ – residue retention; R_-_ – residue removal.

**Table 6:** Data on the interactive effects of tillage practices, crop rotation sequences and residue management systems on the soil chemical indicators scores of the *Haplic Plinthosol* at 0–5 and 5–10 cm depth.

	pH				EC			
	CT		NT		CT		NT	
Treatments	R_+_	R_-_	R_+_	R_-_	R_+_	R_-_	R_+_	R_-_
0–5 cm dep								
MFM	0.95	0.89	0.93	0.93	1.00	1.00	1.00	1.00
MFS	0.98	0.94	0.94	0.94	1.00	1.00	1.00	1.00
MWM	0.87	0.95	0.83	0.90	1.00	1.00	1.00	1.00
MWS	0.98	0.94	0.91	0.96	1.00	1.00	1.00	1.00

5–10 cm								
MFM	0.94	0.86	0.88	0.98	1.00	1.00	1.00	1.00
MFS	0.94	0.91	0.87	0.97	1.00	1.00	1.00	1.00
MWM	0.86	0.92	0.77	0.94	1.00	1.00	1.00	1.00
MWS	0.91	0.92	0.85	0.85	1.00	1.00	1.00	1.00

CT – conventional tillage; NT – no-till; MFM – maize-fallow-maize; MFS – maize-fallow-soybean; MWM – maize-wheat-soybean; MWS – maize-wheat-soybean; R_+_ – residue retention; R_-_ – residue removal.

**Table 7:** Data on the interactive effects of tillage practices, crop rotation sequences and residue management systems on the soil physical indicators and the overall soil quality (SQI) of the *Haplic Plinthosol* at 0–5 and 5–10 cm depth.

	AGS				ρb				SQI			
	CT		NT		CT		NT		CT		NT	
Treatments	R_+_	R_-_	R_+_	R_-_	R_+_	R_-_	R_+_	R_-_	R_+_	R_-_	R_+_	R_-_
0–5 cm depth												
MFM	0.70	0.74	0.63	0.62	0.74	0.89	0.43	0.39	0.88	0.91	0.86	0.83
MFS	0.75	0.87	0.59	0.54	0.73	0.93	0.39	0.42	0.90	0.90	0.84	0.83
MWM	0.65	0.85	0.62	0.63	0.72	0.72	0.53	0.55	0.83	0.88	0.83	0.86
MWS	0.87	0.80	0.61	0.53	0.72	0.86	0.55	0.47	0.89	0.90	0.85	0.82

5–10 cm depth												
MFM	0.36	0.47	0.48	0.40	0.74	0.89	0.43	0.39	0.78	0.82	0.78	0.77
MFS	0.43	0.57	0.45	0.38	0.73	0.93	0.39	0.42	0.82	0.85	0.77	0.77
MWM	0.34	0.35	0.32	0.37	0.72	0.72	0.53	0.55	0.75	0.78	0.75	0.79
MWS	0.36	0.31	0.41	0.38	0.72	0.86	0.55	0.47	0.80	0.79	0.78	0.75

CT – conventional tillage; NT – no-till; MFM – maize-fallow-maize; MFS – maize-fallow-soybean; MWM – maize-wheat-soybean; MWS – maize-wheat-soybean; R_+_ – residue retention; R_-_ – residue removal.

## 2 Experimental Design, Materials and Methods

The soil indicator data was created from the soil analysis of samples from the CA field trial in Phandulwazi, Eastern Cape, South Africa. The design used to set up the field trial for the application of the CA treatments was a split-split plot design. Standard soil procedures were used to generate the raw soil indicator data [2]. Bulk density (ρb) was measured in the field using a coring method as outlined by Hao et al. [4]. A sampler with a known mass and volume was pushed into the soil and three cores were collected from each plot. Excess soil was removed, and soil samples were dried at 105 °C for 24 h and the dry mass were weighed to calculate bulk density. Macro-aggregate stability (AGS) was determined using a modified fast wetting method by Attou et al. [5]. Soil pH was quantified with a pH meter in a 1:2.5 (ν/ν) soil:water suspension as outlined by AgriLASA [6]. Electrical conductivity (EC) was quantified with a conductivity meter in the same suspension used for pH reading after a 1 h settling period [7]. Olsen method was used to extract P in soils as outlined in AgriLASA [6] and P concentrations were quantified using the UV spectrophotometer. Extractable K was extracted using 1 M ammonium acetate at pH 7 as described by AgriLASA [6] and K in the extracts was analyzed using an Inductively Coupled Plasma Emission Spectrograph (ICP–OES) (Varian 710-ES). Total soil carbon (C) was quantified in the air-dried soil samples by dry combustion using the LECO (Truspec CNS analyzer) [8]. Total C in the soil samples was presumed to be equivalent to soil organic carbon (SOC) because the samples had no-carbonate rich minerals. Microbial biomass carbon (MBC) was determined by the modified chloroform fumigation extraction methods [9,10] followed by the dichromate oxidation of carbon [11]. ß-glucosidase activity (BG activity) was computed by colorimetric determination of p-nitrophenyl that is released when 1 g air-dried soil was incubated with 1 mL of p-Nitrophenyl-β-D-glucoside (PNG) and 4 mL of buffer solution (pH 6.0) at 37 °C for 1 h [12]. SMAF scoring algorithms developed by Andrews et al. [13] were used to score the soil indicator data generated from standard soil extraction procedures and experiments. The data scores were processed statistically using JMP statistical package [3].

## Ethics Statements

This work constituted working with soil and did not include work involved with human subjects, animal experiments or data collected from social media platforms.

## CRediT authorship contribution statement

**I. Gura:** Conceptualization, Writing – original draft, Methodology, Formal analysis. **P.N.S. Mnkeni:** Methodology, Writing – review & editing, Supervision. **C.C. Du Preez:** Visualization, Investigation, Supervision. **J.H. Barnard:** Validation, Writing – review & editing.

## Declaration of Competing Interest

The authors declare that they have no known competing financial interests or personal relationships that could have appeared to influence the work reported in this data article.

## Data Availability

Soil indicator data from a Conservation Agriculture trial in Eastern Cape, South Africa (Original data) (Mendeley Data).
